# A combined community- and facility-based approach to improve pregnancy outcomes in low-resource settings: a Global Network cluster randomized trial

**DOI:** 10.1186/1741-7015-11-215

**Published:** 2013-10-03

**Authors:** Omrana Pasha, Elizabeth M McClure, Linda L Wright, Sarah Saleem, Shivaprasad S Goudar, Elwyn Chomba, Archana Patel, Fabian Esamai, Ana Garces, Fernando Althabe, Bhala Kodkany, Hillary Mabeya, Albert Manasyan, Waldemar A Carlo, Richard J Derman, Patricia L Hibberd, Edward K Liechty, Nancy Krebs, K Michael Hambidge, Pierre Buekens, Janet Moore, Alan H Jobe, Marion Koso-Thomas, Dennis D Wallace, Suzanne Stalls, Robert L Goldenberg

**Affiliations:** 1 Department of Community Health Sciences, Aga Khan University, Karachi, Pakistan; 2 Department of Social, Statistical and Environmental Sciences, Research Triangle Institute, Durham, NC, USA; 3 Eunice Kennedy Shriver National Institute of Child Health and Human Development, Bethesda, MD, USA; 4 KLE University’s Jawaharlal Nehru Medical College, Belgaum, India; 5 Department of Pediatrics, University Teaching Hospital, Lusaka, Zambia; 6 Indira Gandhi Government Medical College, Nagpur, India; 7 Moi University School of Medicine, Eldoret, Kenya; 8 Francisco Marroquin University, Guatemala City, Guatemala; 9 Institute for Clinical Effectiveness and Health Policy, Buenos Aires, Argentina; 10 Centre for Infectious Disease Research Zambia, Lusaka, Zambia; 11 University of Alabama at Birmingham, Birmingham, AL, USA; 12 Christiana Care Health Services, Newark, DE, USA; 13 Massachusetts General Hospital for Children, Boston, MA, USA; 14 Indiana University, Indianapolis, IN, USA; 15 University of Colorado, Denver, CO, USA; 16 School of Public Health and Tropical Medicine, Tulane University, New Orleans, LA, USA; 17 Research Triangle Institute, Durham, NC, USA; 18 University of Cincinnati, Cincinnati, OH, USA; 19 American College of Nurse Midwives, Washington, DC, USA; 20 Department of Obstetrics and Gynecology, Columbia University, New York, NY, USA

**Keywords:** Stillbirth, Neonatal mortality, Maternal mortality, Emergency obstetric care

## Abstract

**Background:**

Fetal and neonatal mortality rates in low-income countries are at least 10-fold greater than in high-income countries. These differences have been related to poor access to and poor quality of obstetric and neonatal care.

**Methods:**

This trial tested the hypothesis that teams of health care providers, administrators and local residents can address the problem of limited access to quality obstetric and neonatal care and lead to a reduction in perinatal mortality in intervention compared to control locations. In seven geographic areas in five low-income and one middle-income country, most with high perinatal mortality rates and substantial numbers of home deliveries, we performed a cluster randomized non-masked trial of a package of interventions that included community mobilization focusing on birth planning and hospital transport, community birth attendant training in problem recognition, and facility staff training in the management of obstetric and neonatal emergencies. The primary outcome was perinatal mortality at ≥28 weeks gestation or birth weight ≥1000 g.

**Results:**

Despite extensive effort in all sites in each of the three intervention areas, no differences emerged in the primary or any secondary outcome between the intervention and control clusters. In both groups, the mean perinatal mortality was 40.1/1,000 births (*P* = 0.9996). Neither were there differences between the two groups in outcomes in the last six months of the project, in the year following intervention cessation, nor in the clusters that best implemented the intervention.

**Conclusions:**

This cluster randomized comprehensive, large-scale, multi-sector intervention did not result in detectable impact on the proposed outcomes. While this does not negate the importance of these interventions, we expect that achieving improvement in pregnancy outcomes in these settings will require substantially more obstetric and neonatal care infrastructure than was available at the sites during this trial, and without them provider training and community mobilization will not be sufficient. Our results highlight the critical importance of evaluating outcomes in randomized trials, as interventions that should be effective may not be.

**Trial registration:**

ClinicalTrials.gov NCT01073488

## Background

Complications during labor and delivery are responsible for half the maternal deaths, one-third of stillbirths and a quarter of neonatal deaths occurring each year worldwide [1-8]. These complications, for example, prolonged labor, preeclampsia, infection and hemorrhage, also cause a substantial amount of maternal morbidity and stillbirths and contribute to neonatal mortality and long-term disability [1,2,6]. Antenatal assessment often fails to predict which women will have complications and when these will occur. Their effective management often necessitates urgent, facility-based management of labor by a skilled birth attendant with the ability to provide parenteral medications, carry out procedures, including blood transfusions and cesarean sections, and provide newborn care/resuscitation [4]. However, health care systems in many developing countries struggle to provide skilled attendance and necessary emergency obstetric care [9,10].

Sixty million births per year world-wide currently occur outside facilities, usually without skilled attendance [10]. When an obstetric emergency occurs, women delivering in these settings are at high risk of poor outcomes due to lack of appropriate services. Attempts to reduce perinatal deaths generally focus on medical treatments, community-participatory approaches or health system interventions [5,11-13]. Programs have used various combinations of these interventions [14-22]; however, simultaneous integration of these strategies has not been adequately evaluated to determine whether in aggregate they would reduce perinatal deaths.

A brief discussion of justification for including specific components in our intervention package follows. 1) Community participatory approaches have suggested reductions in maternal/neonatal mortality in Bolivia, India, Bangladesh and Nepal [18-22]. Changing behavior of families and communities during pregnancy and reducing barriers to health service by addressing context-specific delays (for example, birth preparedness, availability of funds, transport mechanisms) have the potential to improve outcomes in populations where most deliveries occur at home or in primary health facilities [23]. 2) Traditional birth attendants (TBAs) remain a major provider of delivery care, especially in settings where mortality rates are highest [24]. Despite the lack of evidence supporting TBA training as a single intervention to reduce mortality [25], some research supports the inclusion of TBAs within an improved health care system focusing on early recognition of obstetric complications and appropriate referral to obstetric care facilities [13-15]. The Global Network’s FIRST BREATH Trial, in which all birth attendants, including TBAs, were taught the World Health Organization’s essential newborn care with emphasis on neonatal resuscitation, suggested a reduction of perinatal mortality associated with this training [26]. 3) Finally, facilities in settings with the worst outcomes are often unable to appropriately implement emergency obstetric and neonatal care packages. Efforts to improve quality of care have focused on in-service training, obstetric simulations/drills or perinatal death audits to improve quality and institute solutions for problems that caused fatalities [27].

This trial tested the hypothesis that teams of health care providers, administrators and local residents can address the limited access to quality obstetric and neonatal care leading to a reduction in perinatal mortality in intervention compared to control locations. These teams worked with their communities and the existing health system to implement a broad package of interventions including community mobilization to establish and sustain mechanisms of transport and payment; training to recognize obstetric emergencies and stabilization and appropriate referral for women delivering at home or in first level care facilities; and improvement of quality of care in existing health facilities.

## Methods

### Study design and setting

This trial was undertaken by the Global Network for Women’s and Children’s Health Research (GN) supported by the Eunice Kennedy Shriver National Institute of Child Health and Human Development [28]. The GN, a multi-country research network with seven research sites in six countries, conducts research to evaluate interventions to reduce maternal and perinatal mortality and morbidity. All seven GN sites participated in the trial, including two in India (Belgaum and Nagpur), and sites in Pakistan, Kenya, Zambia, Guatemala and Argentina. Descriptions of the site populations and resources have been published [28,29].

Details of the trial methods have been described [30]. Briefly, we conducted a community-based, two-arm cluster-randomized trial, including all pregnancies of residents in 106 clusters. A cluster is a distinct geographic area with approximately 500 births per year. Intervention start dates ranged from March to August 2009 and the project intervention period was terminated for all sites on 30 September 2011. Data for the first six months of the implementation were not included in the analysis data set. Thus, the primary outcome period was 18 months (two sites) to 24 months (five sites). We also present outcome data for the last six months of the intervention period and, because a pregnancy registry is ongoing, the full year following cessation of the intervention.

### Subjects

Each site had a pre-existing, independent maternal-newborn health registry system to screen, enroll, and track all pregnant women in the study clusters [28]. Registry administrators enrolled women during pregnancy, obtained informed consent for the trial, and recorded all intervention and control cluster delivery outcomes, including stillbirths and neonatal deaths, and all deaths of pregnant women through 42 days post-delivery or pregnancy termination. Outcomes for all women with births ≥1000 grams and or ≥28 weeks residing within the study cluster for at least four weeks prior to delivery and who consented were included in study. Study site ethics/institutional review boards, partnering US institutions, and RTI International approved the protocol. The trial was registered at ClinicalTrials.gov (NCT ID# NCT01073488).

Based on previously collected data, the 106 study clusters had a mean perinatal death <7 days of age of 40 to 50 per 1,000 deliveries and an intra-class correlation coefficient between 0.005 and 0.01 [26]. Using a two-sided hypothesis test at 5% significance, these 106 clusters, with a minimum of 18 month outcome data, provided a power of at least 80% to detect a 25% reduction in perinatal mortality.

### Randomization and masking

Randomization was performed at the cluster level, stratifying by rates of the primary outcome (stillbirth and early neonatal death) and number of deliveries. The data coordinating center (Research Triangle Institute) produced a computer-generated randomization algorithm which assigned clusters at a 1:1 ratio within each stratum. Because of the nature of the intervention, there was no masking.

### Intervention

**Figure 1:**
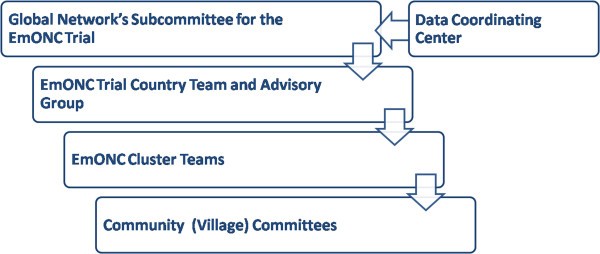
EmONC Study Organization.

Under direction of the GN Steering Committee, a team of GN investigators, trainers with expertise in community mobilization, TBA training and facility quality improvement designed the intervention and provided study oversight (See Figure 1). At each international site, an intervention team of senior health, health system and study personnel, meeting at least monthly, oversaw the project implementation. Trainers with extensive experience in community mobilization, others with experience in training community birth attendants and physicians with expertise in facility staff training were part of the country intervention team and participated in training in the individual clusters. In each study cluster, a cluster team comprising health care providers, local residents and study personnel was formed to develop and implement comprehensive interventions to improve the quality of obstetric and neonatal care. These cluster teams worked within their community and the local health care system to introduce these interventions. Maternal and perinatal mortality audits, facility-level provider training and facility reviews were conducted as quality improvement activities at the facility level. In addition, at the community level, village-level core groups were formed which facilitated community meetings of mothers, family and community birth attendants over the course of the trial. In summary, the cluster teams facilitated a multi-faceted intervention which included the following:

•Community mobilization to establish village-level core groups and to strengthen community capacity to identify and address barriers to obstetric and neonatal care such as recognition of complications and transportation to a facility to manage the complication [18]. Each village-level core group was trained to move through a cycle to organize, plan, explore, act, and to evaluate maternal and perinatal outcomes within their community.

•Home-based Life Savings Skills (HBLSS) training was provided for birth attendants and families to recognize prolonged labor, infection, preeclampsia and hemorrhage, and the use of appropriate stabilization methods that can be employed in homes and in first level care facilities [31-36]; and, improvement of quality of care in existing health facilities through a combination of facility staff Emergency Obstetric and Newborn Care (EMONC) training for clinical care of the major causes of maternal and newborn mortality [37], perinatal and maternal death audits [38,39] and health facility audits [29].

The EmONC trial used a train-the-trainer model for the three main components (HBLSS, community mobilization and facility EMONC training) and the modules which were focused on the major causes of maternal, fetal and neonatal mortality (Table 1). Experienced trainers for each of the three components were identified and these 'master trainers’ with input from other experts, selected and modified the existing curriculum and led the train-the-trainer training as described below:

**Table 1:** Training

**Intervention**	**Purpose**	**Major areas of maternal/newborn mortality addressed**	**Prior research**
**Community mobilization **[18]	To ensure community recognition of pregnancy complications and their importance and to mobilize resources for maternal and newborn care including transportation to an appropriate facility	The community training focused on the major complications that killed women and newborns such as bleeding, preeclampsia/eclampsia, infections, asphyxia and prematurity and the importance of receiving appropriate care for these conditions.	Studies conducted in Nepal, Bangladesh, Pakistan etc. suggested program effectively facilitates community change [19-23]
**ACNM home-based life-savings skills **[31]	To train community birth attendants	Recognition of danger signs, stabilization and referral of mother for obstetric hemorrhage, preeclampsia/eclampsia, sepsis; newborn resuscitation and appropriate referral were emphasized	Studies conducted in Bangladesh, Indonesia and Liberia suggest that the curriculum effectively provides essential skills to nurse midwives and community birth attendants [32-36]
**Jhpeigo emergency obstetric and newborn care curriculum **[37]	To train facility-based health care providers to manage obstetric and neonatal complications and to audit maternal, fetal and neonatal deaths.	The modules addressed the major causes of maternal, fetal and newborn mortality (obstetric hemorrhage, preeclampsia/eclampsia, infection, asphyxia, and prematurity). Death audits, emergency drills, and facility audits were part of the training	EmONC training has been evaluated [15] and the Jhpeigo curriculum tested [38]; evidence for audits to improve care demonstrated [39]

For HBLSS and community mobilization, the training was combined and consisted of two in-country train-the-trainer sessions (an initial two-week period with approximately 70 hours of course work and practicum utilizing the home-based life-saving skills curriculum. The community mobilization/HBLSS training emphasized the Community Action Cycle and the relevant HBLSS modules to identify and perform life-saving measures for the conditions associated with maternal and early newborn mortality (for example, post-partum hemorrhage, preeclampsia/eclampsia, low birth weight newborn care). A second one-week in-country training of trainers and cluster coordinators was held after 12 months. The in-country trainers then trained all of the community birth attendants in the curriculum; these training sessions included an initial three-day training followed by ongoing (minimal of monthly) training and community meetings.

Additionally, the in-country EMONC trainer, usually an experienced obstetric physician, received a three-day course using a train-the-trainer model at a central location utilizing a modified version of the Jhpeigo EMONC curriculum (37). This three-day training emphasized the curriculum addressing post-partum hemorrhage, preeclampsia/eclampsia and emergency preparedness. The in-country trainers then carried out training for the hospitals serving their intervention clusters with the amount of training, including an initial three to five day session to cover the essential elements with additional time dedicated to follow-up training, varying based on local assessment of facility needs. For each of these components, the master trainers participated in central training, followed by in-country training every six months during the 24-month trial period. Each of the training sessions included pre and post-tests to assess knowledge and skills acquisition.

In anticipation that the package of interventions would be better introduced in some clusters than others, we *a priori* created a system for measuring the integrity of the intervention, with credit given for reaching the targets for four intervention measures including monthly cluster team meetings, death audits, village-level core group activities and village-level core groups reaching the 'act’ phase of the community action cycle.

### Study outcomes

The primary outcome was perinatal mortality, defined as the composite of stillbirth and seven-day neonatal mortality per 1,000 births among births occurring at ≥28 weeks gestation or birth weight ≥1000 g. Secondary outcomes included rates of stillbirth (both fresh and macerated), seven-day neonatal mortality, 28-day neonatal mortality and maternal death. Process measures, such as rates of transport to hospital of mothers and newborns and facility delivery, were also determined. Each of these measures was assessed in both intervention and control clusters through the registries, with registry teams distinct from those implementing the interventions. We also collected extensive data on the intervention itself including number and type of cluster and community meetings, death audits and providers trained.

### Statistical analyses

Data were entered at each study site with inter- and intra-data edits and consistency checks performed. The Data Monitoring Committee reviewed the data for safety and efficacy. Generalized estimating equations (GEE) extensions of a log-binomial for multivariate logistic regression model that accounted for the study design strata and correlation between outcomes in the same cluster tested for differences in the primary outcome. Secondary outcome analyses were conducted using GEE extensions of either log-binomial or robust Poisson regression models for binary outcomes and clustered multinomial logistic regression model extensions for ordinal outcomes. All analyses were performed using SAS version 9.3 (SAS Institute, Cary, NC), with the exception of the multinomial logistic regression models, which were performed using SUDAAN 11.

### Ethics approval and consent

The Ethics Review Committees of each participating institution and the data coordinating center (RTI International) all approved the study protocol. Informed consent was obtained from all women who participated in the study.

### Role of funding source

This trial was funded by grants from the US National Institutes of Health (NIH). The NIH program officers (LLW, MKT) participated in the protocol development and study monitoring, and reviewed the manuscript.

## Results

**Figure 2:**
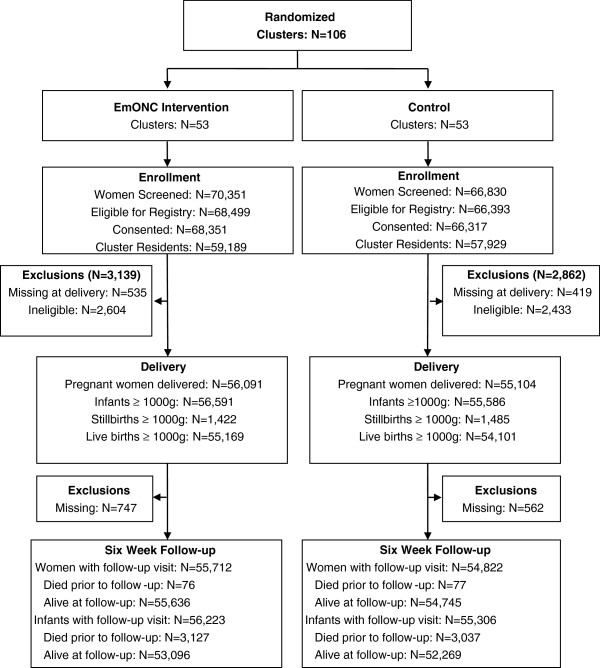
Consort diagram.

A total of 106 clusters, ranging from 6 in Argentina to 24 in Pakistan, were randomized, with 53 in each treatment group (Figure 2). A total of 70,351 pregnant women were screened in the intervention clusters and 66,830 in the control clusters; of these, 59,189 (84%) and 57,929 (87%) women were eligible and consented in the intervention and control groups, respectively. Those women excluded, for the most part, did not reside in the cluster for a full four weeks prior to delivery. An additional 3,139 (4.5%) and 2,862 (4.3%) deliveries in intervention and control clusters were excluded at delivery due to missing data or other ineligibility criteria. Outcomes were obtained for >99% of eligible women at six-weeks post-delivery (55,712 and 54,822 in the intervention and control clusters, respectively).

Demographic characteristics of the intervention and control participants are shown in Table 2. Overall, almost one-third of women had no formal schooling and 5% had university-level education. Approximately 12% of the mothers were <20 years of age and 83% were 20 to 35 years old. In both groups, about 30% of the women were primiparous; over 12% had more than four prior pregnancies. Table 2 also shows the birthweight, gestational age, and gender distributions. Although there were small differences, none were statistically significant at *P* <0.05.

**Table 2:** Maternal and neonatal characteristics

	**Group**	
	**Treatment**	**Control**
Total clusters, N	53	53
Deliveries by country, N		
Argentina (6 clusters)	2,483	2,717
Guatemala (10 clusters)	6,898	5,405
Kenya (16 clusters)	5,962	7,401
Zambia (10 clusters)	6,455	7,423
Belgaum, India (20 clusters)	14,317	12,585
Nagpur, India (20 clusters)	6,535	5,966
Pakistan (24 clusters)	13,080	13,338
Mothers (N)	55,730	54,835
Maternal education, N (%)	55,325	54,524
No formal schooling	16,781 (30.3)	16,245 (29.8)
Primary	22,016 (39.8)	20,485 (37.6)
Secondary	13,476 (24.4)	14,879 (27.3)
University +	3,052 (5.5)	2,915 (5.3)
Maternal age, N (%)	55,602	54,757
< 20	6,870 (12.4)	6,838 (12.5)
20 to 35	46,320 (83.3)	45,339 (82.8)
> 35	2,412 (4.3)	2,580 (4.7)
Parity, N (%)	55,619	54,754
0	16,986 (30.5)	16,663 (30.4)
1 to 4	31,687 (57.0)	30,949 (56.5)
> 4	6,946 (12.5)	7,142 (13.0)
Infants ≥1000 g, N	56,223	55,306
Birth weight, N (%)	56,223	55,306
1000 to 1499 g	687 (1.2)	739 (1.3)
1500 to 2499 g	6,170 (11.0)	5,431 (9.8)
≥2500 g	49,366 (87.8)	49,136 (88.8)
Gestational age, N (%)	56,128	55,197
Term	51,681 (92.1)	51,409 (93.1)
Preterm	4,447 (7.9)	3,788 (6.9)
Gender, N (%)	56,174	55,233
Male	28,989 (51.6)	28,964 (52.4)
Female	27,185 (48.4)	26,269 (47.6)

Table 3 shows the number of interventions applied in the treatment clusters by site, emphasizing the great amount of work done by the site and cluster teams. The 53 intervention cluster teams (one per cluster) led more than 50,000 cluster-level activities, including meetings, trainings and other community sensitization activities. The specific activities included facility training (n = 1,309), HBLSS training (n = 26,623), community mobilization training (n = 21,060) and other meetings (n = 1,441). There were a total of 304 facility reviews; on average, each facility was reviewed more than four times during the trial. There were 5,039 facility death audits. In each site, more death audits were completed than there were study deaths because facility audits included deaths of women and their newborns from non-study areas. Village-level core groups went through the community action cycle stages (organize, explore, plan, act and evaluate) generally in sequential order; however, due to occasional division of one into two core groups, a few appear to start the sequence at mid-cycle. The primary issues identified during these activities were difficulty recognizing that the mother/baby had a problem requiring treatment, lack of transportation and lack of funds to pay for facility care (data not shown). Altogether, 96.7% of the village-level core groups reached the act stage by the end of the trial. Using these data and the *a priori* criteria for implementing the intervention, 37.7% of the clusters met two or fewer criteria, 32.1% of the clusters met three criteria, and 30.2% met all four criteria.

**Table 3:** The number of activities in intervention clusters by study site and where appropriate, means and percentages

	**Total**	**Argentina**	**Guatemala**	**Kenya**	**Zambia**	**Belgaum, India**	**Nagpur, India**	**Pakistan**
Cluster team-led activities								
Total activities, N	50,433	322	1,916	5,703	7,941	3,272	13,053	18,226
Facility training	1,309	94	115	183	27	497	80	313
HBLSS training	26,623	101	559	2,898	4054	698	7,569	10,744
Community training or meeting	21,060	111	1,095	2,595	3814	2,059	5,404	6,054
Other activity	1,441	16	147	99	46	18	0	1,115
Health facility provider training								
Providers trained, N	1,459	76	364	140	302	295	209	73
Health facility reviews								
Total facilities, N	304	3	23	22	9	69	130	48
Average reviews per facility,	4.1 (1.0)	5.0 (0.0)	4.8 (0.4)	3.5 (0.7)	5.0 (0.0)	4.6 (0.7)	3.5 (0.8)	4.3 (1.3)
Mean (SD)								
Facility death audits,	5,039 (126.3)	86 (103.6)	382 (117.9)	180 (84.1)	309 (110.0)	1,287 (104.6)	321 (92.5)	2,474 (163.6)
N (% of total deaths)								
Core groups								
Core groups, N	3,721	7	50	231	160	667	1,028	1,578
Total meetings, N	119,200	187	1,681	9,960	8,861	33,802	11,949	52,760
Community mobilization stage reached by core groups, N (%)								
Organize	3,396 (91.3)	3 (42.9)	50 (100.0)	216 (93.5)	159 (99.4)	665 (99.7)	932 (90.7)	1,371 (86.9)
Explore	3,536 (95.0)	5 (71.4)	49 (98.0)	231 (100.0)	159 (99.4)	666 (99.9)	886 (86.2)	1,540 (97.6)
Plan	3,486 (93.7)	6 (85.7)	44 (88.0)	231 (100.0)	160 (100.0)	661 (99.1)	828 (80.5)	1,556 (98.6)
Act	3,600 (96.7)	6 (85.7)	49 (98.0)	220 (95.2)	160 (100.0)	661 (99.1)	1,006 (97.9)	1,498 (94.9)
Evaluate	2,647 (71.1)	3 (42.9)	46 (92.0)	160 (69.3)	54 (33.8)	660 (99.0)	441 (42.9)	1,283 (81.3)

Table 4 summarizes the indicators of the antenatal and delivery care by intervention and control groups after the trial’s initiation. In both arms, almost 60% of women attended at least one antenatal class. More than three-fifths of women in both groups reported having access to emergency transport, if necessary, for delivery, and the rate of facility delivery in both groups was 65%. In the intervention group, 61.3% of women reported access to emergency funds in case a facility delivery was needed, compared to 56.8% in the control group and 87.1% of women in the intervention group had identified a birth attendant prior to delivery compared to 84.6% in the control group. However, neither of these differences rose to statistical significance.

**Table 4:** Indicators of quality of care by treatment group

	**Group**		
	**Intervention**	**Control**	**P-value**
Clusters, N	53	53	
Mothers with infants ≥1000 g, N	55,730	54,835	
Indicators of quality care, Mean (95% CI)			
Attended at least one antenatal care class	59.7 (55.8, 63.7)	59.7 (55.8, 63.5)	0.6687
Access to an emergency fund/plan for hospital delivery	61.3 (54.3, 68.3)	56.8 (50.2, 63.4)	0.3541
Birth attendant identified prior to birth	87.1 (82.6, 91.7)	84.6 (79.5, 89.7)	0.3269
Identified birth attendant present at birth	71.0 (66.8, 75.2)	71.8 (66.5, 77.2)	0.8045
Transport identified prior to birth	62.0 (55.5, 68.6)	62.3 (56.4, 68.1)	0.9570
Received tetanus toxoid vaccine	87.9 (86.3, 89.4)	87.9 (86.4, 89.5)	0.2808
Received prenatal vitamins/iron	89.6 (87.9, 91.4)	89.8 (88.1, 91.4)	0.1792
Received syphilis test	18.5 (14.9, 23.0)	19.5 (15.4, 24.8)	0.5237
Received HIV test	71.2 (69.2, 73.2)	71.3 (69.1, 73.4)	0.9457
Cesarean section (of all deliveries)	11.7 (10.1, 13.2)	11.9 (10.4, 13.4)	0.5022
Physician or nurse/midwife deliveries	65.0 (62.5, 67.6)	64.8 (61.9, 67.6)	0.7053
Traditional birth attendant deliveries	32.4 (26.3, 38.4)	32.5 (26.2, 38.9)	0.8998
Hospital deliveries	38.6 (32.4, 44.7)	40.3 (33.6, 47.0)	0.4801
Clinic/health center deliveries	24.6 (20.4, 28.8)	24.0 (19.3, 28.7)	0.9131
Clean razor was used to cut cord	87.6 (86.0, 89.3)	87.5 (85.9, 89.2)	0.4844
Birth attendant used new gloves	95.5 (94.3, 96.6)	95.7 (94.8, 96.6)	0.5934
Newborn resuscitated with bag and mask	2.6 (1.9, 3.3)	3.1 (2.1, 4.1)	0.3769

Table 5 first shows the primary outcome of perinatal mortality and the secondary outcomes for the 18 to 24 months of the intervention. In both the intervention and the control clusters, the mean perinatal mortality was 40.1/1,000 births (*P* = 0.9996). None of the secondary outcomes differed significantly between groups. Next, to determine whether differences developed only late in the project, we evaluated each outcome in the last six months of the intervention. No significant differences in outcome emerged. Finally, we examined the primary outcomes for intervention compared to control clusters in the 12 months post-trial and found no significant differences in any outcome measure. We also compared the outcomes in the better performing intervention clusters to their controls for the entire intervention time period, and for the last six months of the intervention (Table 6). There were no significant differences in outcomes in either time period.

**Table 5:** Neonatal and maternal mortality outcomes by treatment for all clusters

	**Group**		
	**Intervention**	**Control**	**P-value**
Clusters, N	53	53	
Outcomes for full intervention period			
Births ≥1000 g, N	56,223	55,306	
Outcomes - Mean (95% CI)			
Perinatal mortality (<7 days) (Rate/1,000)	40.1 (37.3, 42.9)	40.1 (37.4, 42.8)	0.9996
Stillbirths (Rate/1,000)	21.8 (19.8, 23.9)	22.6 (20.5, 24.6)	0.6177
Fresh stillbirth (Rate/1,000)	15.2 (13.4, 17.0)	15.5 (14.0, 17.0)	0.8082
Early neonatal mortality (<7 days) (Rate/1,000)	18.7 (17.3, 20.1)	18.2 (16.6, 19.7)	0.5950
Neonatal mortality (<28 days) (Rate/1,000)	23.8 (22.0, 25.5)	22.5 (20.6, 24.5)	0.3362
Fresh stillbirths + neonatal deaths <7 days (Rate/1,000)	33.8 (31.2, 36.3)	33.4 (31.2, 35.5)	0.8105
Maternal mortality (<42 days) (Rate/100,000)	125.1 (97.7, 160.2)	130.9 (104.5, 163.9)	0.7321
Outcomes for final six months of intervention period			
Births ≥1,000 g, N	15,412	15,180	
Outcomes - Mean (95% CI)			
Perinatal mortality (<7 days) (Rate/1,000)	39.6 (35.8, 43.4)	41.4 (37.4, 45.5)	0.5043
Stillbirths (Rate/1,000)	21.6 (18.8, 24.4)	22.9 (19.7, 26.2)	0.5491
Fresh stillbirth (Rate/1,000)	15.2 (12.7, 17.7)	14.5 (12.2, 16.8)	0.6826
Early neonatal mortality (<7 days) (Rate/1,000)	18.7 (16.7, 20.8)	19.0 (16.8, 21.3)	0.8458
Neonatal mortality (<28 days) (Rate/1,000)	23.4 (21.0, 25.8)	22.9 (20.3, 25.6)	0.7822
Fresh stillbirths + neonatal deaths <7 days (Rate/1,000)	33.4 (30.1, 36.8)	33.4 (30.3, 36.4)	0.9734
Maternal mortality (<42 days) (Rate/100,000)	109.1 (64.5, 184.5)	78.3 (44.0, 139.3)	0.2799
Outcomes for 12-months post-intervention period			
Births ≥1,000 g, N	27,852	26,356	
Outcomes - Mean (95% CI)			
Perinatal mortality (<7 days) (Rate/1,000)	37.9 (34.0, 41.7)	36.6 (33.2, 40.0)	0.6202
Stillbirths (Rate/1,000)	21.7 (19.2, 24.2)	21.1 (18.9, 23.4)	0.7406
Fresh stillbirth (Rate/1,000)	14.8 (12.7, 16.9)	14.1 (12.2, 16.0)	0.6271
Early neonatal mortality (<7 days) (Rate/1,000)	16.4 (14.5, 18.4)	15.9 (13.8, 18.0)	0.7136
Neonatal mortality (<28 days) (Rate/1,000)	21.4 (19.0, 23.8)	20.0 (17.5, 22.5)	0.4311
Fresh stillbirths + neonatal deaths <7 days (Rate/1,000)	31.3 (27.7, 34.9)	29.7 (26.6, 32.9)	0.5126

**Table 6:** Neonatal and maternal outcomes for well performing intervention clusters and control clusters in the same strata for the entire intervention and restricted to the last six months of the intervention

	**Group**		
	**Well performing intervention clusters**	**Control clusters same strata**	**P-value**
Clusters, N	33	36	
Outcomes for full intervention period			
Births ≥1,000 g, N	40,897	42,662	
Primary outcome - Mean (95% CI)			
Perinatal mortality (<7 days) (Rate/1,000)	40.6 (37.0, 44.1)	40.4 (37.0, 43.7)	0.9404
Secondary outcomes - Mean (95% CI)			
Stillbirths (Rate/1,000)	21.8 (19.2, 24.5)	23.3 (21.0, 25.6)	0.4087
Fresh stillbirth (Rate/1,000)	14.9 (12.5, 17.3)	15.7 (14.0, 17.4)	0.5504
Early neonatal mortality (<7 days) (Rate/1,000)	19.2 (17.6, 20.8)	17.8 (16.2, 19.3)	0.2095
Neonatal mortality (<28 days) (Rate/1,000)	24.6 (22.6, 26.7)	21.9 (20.0, 23.7)	0.0516
Fresh stillbirths + neonatal deaths <7 days (Rate/1,000)	33.9 (30.6, 37.1)	33.2 (30.4, 36.0)	0.7365
Maternal mortality (<42 days) (Rate/100,000)	103.2 (71.7, 148.5)	108.5 (76.0, 154.8)	0.7425
Outcomes for final six months of intervention period			
Births ≥1,000 g, N	10,514	10,568	
Primary outcome - Mean (95% CI)			
Perinatal mortality (<7 days) (Rate/1,000)	38.1 (33.6, 42.6)	41.3 (36.5, 46.1)	0.3666
Secondary outcomes - Mean (95% CI)			
Stillbirths (Rate/1,000)	21.3 (18.0, 24.6)	23.2 (19.4, 27.0)	0.4976
Fresh stillbirth (Rate / 1,000)	14.4 (11.3, 17.5)	13.3 (10.9, 15.8)	0.6080
Early neonatal mortality (<7 days) (Rate/1,000)	17.7 (14.8, 20.6)	18.7 (15.7, 21.7)	0.6118
Neonatal mortality (<28 days) (Rate/1,000)	23.0 (19.3, 26.8)	22.8 (19.1, 26.5)	0.9220
Fresh stillbirths + neonatal deaths <7 days (Rate/1,000)	31.4 (27.3, 35.6)	32.1 (28.7, 35.5)	0.8153

## Discussion

In geographic areas with high maternal and perinatal mortality in seven sites in six countries, we found that a multipronged intervention that included: 1) community mobilization and birth attendant education focusing on birth planning and transportation to a hospital; 2) birth attendant recognition of complications, stabilization and appropriate, timely referral to a hospital; and 3) hospital staff training focusing on appropriate and timely management of medical complications did not reduce perinatal mortality.

We have considered the potential reasons why our efforts did not achieve the hypothesized outcomes. One possibility is that we had the right group of interventions to achieve an important improvement in outcomes, but that the intervention was not carried out sufficiently well in enough clusters to impact either the process measures or the outcomes. However, based on our ongoing monitoring systems, we documented that the intervention components were generally implemented with high fidelity. The very large number of activities documented plus the fact that there was no improvement in outcomes even among the best performing clusters, suggests this was not the case. Another possibility is that our chosen package of interventions was not implementable with the resources or time allowed for this project. However, substantial resources were allocated to the intervention (each study site had a budget over the two years of approximately $500,000 USD or on average about $60,000 USD per intervention cluster) and many of the clusters achieved substantial compliance in most components of the intervention. That there were no observable improvements in outcome in the final six months of the intervention or even in the year after the intervention ceased, suggests that insufficient time was not responsible for the lack of observed improvement in outcome. Another possibility is that although we had three intervention components, most sites appeared to give more attention to community mobilization and community birth attendant training and less to hospital staff training. Since a well-functioning hospital and a trained, motivated staff seem crucial for achieving the level of mortality reduction hoped for in this study, it may be that hospital training was insufficient. However, a substantial amount of hospital training occurred, and potential areas for improvement were made apparent to the hospital administrators and staff by the facility and mortality audits.

A more likely explanation for the lack of improvement in outcomes in the intervention clusters is that the deficiencies in the health systems were beyond potential improvement by our package of interventions. Without appropriate complementary efforts by the ministries of health or other agencies to strengthen the health care infrastructure at referral facilities to ensure availability of skilled personnel and access to comprehensive emergency obstetric care including appropriate essential medications, supplies, and equipment, it is likely that interventions predominantly focusing on community mobilization and birth attendant training alone are insufficient to achieve the hoped-for results [22-25,29]. Community mobilization or birth attendant training might only help to reduce mortality beyond a certain level if there is a concurrent improvement in the capacity for managing obstetric and neonatal emergencies. Our study suggests that the weaknesses of the delivering facilities and health systems in the participating sites, including the lack of essential medications, supplies and equipment, were not adequately addressed by the three-pronged strategy used in this trial [29].

This study had a number of strengths including the participation of seven sites in six countries and the large number of clusters. The populations in the intervention and the control arms were similar. A further strength is that the study was performed in locations which had ongoing pregnancy registries with excellent follow-up of mothers and infants, and with registry personnel distinct from trial staff, reducing potential for bias. That data collection continued for a full year after the intervention ceased is a further strength. A potential weakness was that all the clusters did not achieve complete implementation, although a substantial amount of work was done in every cluster. Another potential weakness was that because the intervention could not be blinded, some of the control clusters may have adopted a portion of the trial interventions. We do not think that contamination occurred frequently, but cannot rule out this possibility.

## Conclusions

Our results are of major public health importance. First, rolling out programs similar to the one tested here without formal testing of their effectiveness will not help to improve pregnancy-related outcomes nor achieve progress toward Millennium Development Goals 4 and 5 to reduce maternal and child mortality. Second, we believe that substantial attention to creating a system of maternal and neonatal care with adequate supplies and infrastructure, in addition to training of existing personnel, is needed to achieve substantial improvements in pregnancy outcomes - specifically systems that include appropriate access to delivery facilities for all women, facilities with sufficient equipment and access to essential medications, and especially a well-trained and motivated staff with a high degree of skill in treating obstetric and neonatal emergencies. Our results suggest that in many low-resource settings, it may take substantial resources and time to create a sustainable and functioning maternal and newborn care system to accomplish this goal than were available for the current project.

## Abbreviations

EMONC: Emergency obstetric and newborn care; GEE: Generalized estimating equations; GN: Global network for women’s and children’s health; HBLSS: Home-based life savings skills; NIH: National institutes of health; TBA: Traditional birth attendant; USD: United States dollar.

## Competing interests

The authors declare that they have no competing interests.

## Authors’ contributions

OP, RLG, EMM, LLW, SS, SSG, EC, AP, FE, AG, FA, BK, HM, AM, WAC, PLH, RJD, EKL, NK, KMH, AJ, PB, MKT and SS conceived the study and developed the study protocol, contributed to the study design, the data collection and monitoring for the study. OP, RLG, EMM, JM and DDW performed data analyses and initial interpretation of results. OP and RLG wrote the initial draft of the manuscript. All of the authors reviewed the data, and reviewed and approved the final manuscript.

## Pre-publication history

The pre-publication history for this paper can be accessed here:

http://www.biomedcentral.com/1741-7015/11/215/prepub
